# Safety, tolerability, and pharmacokinetics of a 2 g subcutaneous dose of ceftriaxone as an alternative to intravenous delivery

**DOI:** 10.1128/aac.01081-25

**Published:** 2025-11-24

**Authors:** Henco Nel, Fionnuala Murray, Okhee Yoo, Matthew Rawlins, Edward Raby, Madhu Page-Sharp, Brioni Moore, Sam Salman, Laurens Manning

**Affiliations:** 1 Department of Infectious Diseases, Fiona Stanley Fremantle Hospitals Group 821494, Murdoch, Western Australia, Australia; 2 Wesfarmers Centre of Vaccines and Infectious Diseases, Telethon Kids Institute, University of Western Australia 2720, Perth, Western Australia, Australia; 3 Pharmacy, School of Allied Health, The University of Western Australia 2720, Perth, Western Australia, Australia; 4 Institute for Paediatric Perioperative Excellence, The University of Western Australia 2720, Perth, Western Australia, Australia; 5 Centre for Optimisation of Medicines, School of Allied Health, University of Western Australia 2720, Perth, Western Australia, Australia; 6 Department of Pharmacy, Fiona Stanley Fremantle Hospitals Group 821494, Murdoch, Western Australia, Australia; 7 Department of Microbiology, PathWest Laboratory Medicine, Fiona Stanley Hospital 418838, Murdoch, Western Australia, Australia; 8 Curtin Medical School, Curtin University 614117, Bentley, Western Australia, Australia; 9 Clinical Pharmacology and Toxicology Unit, PathWest Laboratory Medicine, Fiona Stanley Hospital 418838, Murdoch, Western Australia, Australia; 10 Medical School, Faculty of Health and Medical Sciences, The University of Western Australia 2720, Crawley, Western Australia, Australia; Providence Portland Medical Center, Portland, Oregon, USA

**Keywords:** subcutaneous antibiotics, ceftriaxone, pharmacokinetics, safety, tolerability

## Abstract

Subcutaneous delivery of antibiotics is a practical alternative to intravenous administration. Ceftriaxone is commonly used for a variety of infections with limited data on the safety and pharmacokinetics of a 2 g subcutaneous dose. This was a prospective, self-controlled cross-over study in 20 stable inpatients receiving ceftriaxone for their infection. Following an intravenous dose, participants received a single dose of 2 g subcutaneous ceftriaxone, in 50 mL normal saline via gravity feed. Capillary dried blood spots were collected at baseline, 1, 2, 4, 8, and 24 hours following the subcutaneous and intravenous doses. Pain scores and infusion site reactions (edema/erythema) were assessed. Ceftriaxone concentrations were measured using a validated liquid chromatography-tandem mass spectrometry assay. A population pharmacokinetic model was developed using nonlinear mixed-effects modeling. The highest median (interquartile range) pain score within the first 2 hours following infusion of a subcutaneous dose of 2 g ceftriaxone was 2.5 (1–4). All participants were pain-free 4 hours after the infusion. The estimated bioavailability was 95.7% (95% bootstrap interval 90.3–99.5). Compared with intravenous, subcutaneous administration resulted in lower peak and comparable trough concentrations. The probability of target attainment for free drug concentrations was similar to intravenous administration for most common infections in hospitalized patients. Subcutaneous administration of 2 g ceftriaxone is well tolerated and has a comparable pharmacokinetic profile relative to intravenous dosing in non-critically ill patients with severe infections.

**IMPORTANCE:**

This prospective, self-controlled cross-over design study demonstrates that subcutaneous administration of 2 g ceftriaxone appears safe and well tolerated with a comparable pharmacokinetic profile relative to intravenous dosing in non-critically ill patients with severe infections.

**CLINICAL TRIALS:**

This study was registered at ACTRN12624000692538.

## INTRODUCTION

Ceftriaxone is a third-generation parenteral cephalosporin used for treatment of hospitalized patients with a variety of infections including severe community-acquired pneumonia, urinary tract infections, intra-abdominal infections, and meningitis (1–3). It is also commonly used in outpatient parenteral antimicrobial therapy (OPAT) settings due to its safety profile and once-daily dosing for most indications (4, 5).

Ceftriaxone is typically administered intravenously (IV). Data supporting intramuscular (IM) administration are provided in the product information sheet, but due to injection-related pain, this route is infrequently used (6). By contrast, subcutaneous (SC) administration is not currently recommended in clinical guidelines or by the manufacturers but may be better tolerated and have favorable pharmacokinetics.

Administration of SC antibiotics has been practiced in Europe, particularly in France, for many years (4, 7, 8). The SC route has garnered increasing attention in recent years as a practical alternative to IV delivery, with increasing evidence in the literature supporting its safety and efficacy for various antibiotics (4, 9–13). This route of administration offers an attractive alternative where IV access is difficult or not preferred. Previous research has also indicated a preference for SC administration over IV from a patient perspective (14).

Previous studies have demonstrated that SC ceftriaxone may be a feasible alternative to IV administration (4, 15). Bioavailability of 95%–100%, together with lower peak and higher trough concentrations, has been reported, providing theoretical pharmacokinetic-pharmacodynamic (PK/PD) advantages over IV administration (15). To date, most of these studies have demonstrated the safety and PK profile of a 1 g ceftriaxone dose. SC administration of a 2 g dose has not been systematically evaluated, and at least one case of severe skin necrosis associated with this higher dose has been described (16).

Interest in formal evaluation of the safety and tolerability of a 2 g dose of SC ceftriaxone has been prompted by updates to national and international guidelines, which have replaced 1 g with a 2 g dose for respiratory, urinary, intra-abdominal, and musculoskeletal infections (2, 17–19).

Building on our recent studies for other SC antibiotics, we hypothesized that ceftriaxone 2 g delivered SC would be well tolerated in hospitalized adults with severe infections and have good bioavailability, resulting in a comparable or improved PK profile (9, 10).

## MATERIALS AND METHODS

### Study design

We conducted a prospective, self-controlled cross-over trial, with PK data collected for SC and IV administration for each participant. The primary objective was to establish tolerability of SC administration of 2 g ceftriaxone using a numerical rating score (NRS) for pain, local erythema, and local edema. The secondary objective was to assess if SC ceftriaxone 2 g had equivalent bioavailability to IV. This study was approved by the South Metropolitan Health Service Ethics Committee (RGS6590) and prospectively registered (Australia and New Zealand Clinical Trials Registry; ACTRN12624000692538). All participants provided written informed consent.

### Participants

Eligible participants recruited into the study were aged 18 years or older, admitted to Fiona Stanley Hospital, Western Australia, and being treated with IV ceftriaxone 2 g as part of their infection management plan. Participants had to be clinically stable at the time of enrollment, defined as not requiring intensive care/high dependency unit admission or having had a medical emergency team (MET) call within the preceding 24 hours. Full exclusion criteria are provided (Table S1). Sample size calculation for detection of 10% lower bioavailability of SC dosing compared to IV dosing and to demonstrate equivalent bioavailability between the two routes of administration, based on FDA/EMA guidance for bioequivalence, would require 20 participants with complete data sets (20).

### Study interventions

Following at least one dose of IV ceftriaxone 2 g, capillary dried blood spots (DBS) were collected from stable participants after an IV dose to establish individual PK parameters. Participants then received a single SC infusion of ceftriaxone 2 g, diluted in 50 mL of normal saline delivered into the tissues of the lower anterior abdominal wall via gravity feed over approximately 30 minutes, using a 24-gage SC catheter (BD Saf-T-Intima, BD Medical, Mississauga, ON, Canada). A saline flush (10–20 mL) was given at the end of the infusion via gravity feed over 5–10 minutes.

### PK sampling

For both IV and SC dosing, DBS were collected from finger prick samples immediately prior to dosing and 1, 2, 4, 8, and 24 hours post commencement of the dose. Additional DBS were collected at 12 hours for participants receiving twice-daily dosing. Baseline hematocrit, serum creatinine, and albumin measures were obtained from blood tests collected as part of routine clinical care on the day of dosing. Creatinine clearance (CrCl) was subsequently calculated using the Cockcroft-Gault equation.

The DBS cards were then air-dried for 1 hour before being placed into an airtight foil bag with two desiccant sachets and subsequently stored at −80°C until transfer to the analytical laboratory on dry ice. Ceftriaxone concentrations from DBS were measured using a validated liquid chromatography-tandem mass spectrometry (LC-MS/MS) assay with a limit of quantification of 0.1 mg/L, as previously described (21, 22).

### Tolerability and safety

Tolerability of SC administration was assessed at the same time points as DBS sampling using the NRS for pain assessment, with scores from 0 to 10, where 0 represents “no pain” and 10 represents the “worst pain imaginable” (23). Skin at the SC infusion site was evaluated for local erythema and edema in all patients by a single clinician (HN). Erythema was scored on a 0-to-4-point scale, with 0 representing “No Erythema” and 4 representing “Severe Erythema to Slight Eschar Formation.” Edema was scored using a 0-to-4-point scale, with 0 representing “No Oedema” and 4 representing “Severe Oedema (raised more than 1  mm and beyond exposure area).”

### Population pharmacokinetics modeling

A population PK model was developed using NONMEM (v7.5.1, ICON Development Solutions, Ellicott City, MD, USA), with a Gfortran 4.6.0 compiler, supported by Perl-Speaks-NONMEM (PsN) and Pirana (24). Parameter estimation was performed using the first-order conditional estimation method with interaction. Data management was conducted using R (v4.1.3, R Foundation for Statistical Computing, Vienna, Austria), Microsoft Excel, and Power BI Desktop (v2.126.1261.0). Model diagnostics and graphical evaluations were carried out using the xpose4 package.

The statistical significance of adding parameters to nested models was assessed using the objective function value (OFV). A decrease in OFV (ΔOFV) of 3.84 between two nested models differing by one parameter was considered statistically significant at the 5% level (*P* = 0.05). Model selection was further guided by graphical analyses, including goodness-of-fit plots, simulation-based prediction-corrected visual predictive checks (VPCs), and evaluation of credible parameter estimates with satisfactory precision.

**Fig 1:**
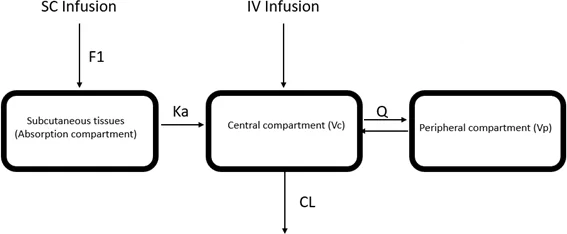
Schematic representation of the two-compartment PK model for ceftriaxone. CL, clearance; Ka, subcutaneous absorption rate constant; Vc, central volume of distribution; Q, intercompartment clearance; Vp, peripheral volume of distribution.

The custom ADVAN6 subroutine was utilized, modeling SC infusion as input into the depot compartment, with bioavailability and absorption rates to the central compartment (Fig. 1).

Both one- and two-compartment models incorporating first-order elimination were evaluated. Interindividual variability (IIV) in population PK parameters was estimated, and residual error was assessed using additive, proportional, and combined models.

Transit, zero-order, and lag absorption models were also explored. *A priori*, allometric scaling with exponents of 0.75 and 1 was applied to body weight for all clearance and volume of distribution terms, respectively. After establishing a satisfactory structural model, covariate relationships (e.g., albumin, CrCL, bilirubin, age, and sex on clearance [CL]; albumin, age, hemoglobin, and hematocrit on volume of distribution [V]; and body mass index [BMI] on absorption rate constant [KA]) were assessed using a forward inclusion method (*P* < 0.05; ΔOFV > 3.84; 1 df), followed by backward elimination (*P* < 0.01; ΔOFV > 6.63; 1 df).

The final model of DBS (whole blood) concentrations was used for simulation. Simulated whole blood concentrations were converted to free plasma concentrations using a previously reported blood cell partitioning ratio and the unbound fraction in plasma (21), along with the median hematocrit value (0.37) from the present data set:

C_u_ = Fu/(H×ρ+(1 H))×C_DBS_

where Cu is the unbound plasma concentration, fu is the unbound fraction in plasma, H is the hematocrit, ρ is the blood cell partitioning ratio, and C_DBS_ is the blood concentration.

Simulations at steady-state concentrations were employed to determine the probability of target attainment (PTA). For each dose administration, simulation was performed on a population of 1,000 individuals, with covariates uniformly distributed based on the demographics of the study population.

Target attainment was defined as unbound ceftriaxone concentrations exceeding the minimum inhibitory concentrations (MIC) for >40% of the time between injections. The PTA was calculated as the proportion of patients achieving this target across different administration routes and over a range of clinically relevant MIC values. Notably, an MIC of 1 mg/L corresponds to the Clinical and Laboratory Standards Institute (CLSI) breakpoints for *Streptococcus pneumoniae* and Enterobacterales (25).

## RESULTS

### Baseline characteristics

A total of 121 participants were screened, with 22 eligible participants recruited. Two participants dropped out before receiving a SC dose because their treating team switched them to oral antibiotics.

Ceftriaxone indications were varied (Table 1). Four participants were being treated for complicated urinary tract infections, three for liver abscess, two for ascending cholangitis, and two for epididymo-orchitis. Other indications included an infected intra-abdominal collection, skin and soft tissue infection, prosthetic joint infection, prosthetic valve infective endocarditis, community-acquired pneumonia, salmonella bacteremia, streptococcal bacteremia, tubo-ovarian abscess, and a maxillary sinus abscess with intra-cranial extension.

**TABLE 1:** Patient demographic details

Parameter	Result for parameter (*n* = 20)
Male sex, n (%)	14 (70%)
Age, years (IQR)	64 (44–77)
Infection source, n (%)	
Genito-urinary	6 (30%)
Intra-abdominal	6 (30%)
Skin and soft tissue	1 (5%)
Bone and joint	1 (5%)
Infective endocarditis	1 (5%)
Primary bacteremia	2 (10%)
Respiratory	1 (5%)
Gynecological	1 (5%)
Central nervous system^*a*^	1 (5%)
Dosing frequency (daily), n (%)	19 (95%)
BMI, kg/m^2^ (IQR)	27 (23–32)
Creatinine, µmol/L (IQR)	78 (62–88)
Albumin, g/L (IQR)	35 (31–36)
Hematocrit, % (IQR)	38 (29–40)

^*a*^ Sinus abscess with intra-cranial extension.

Fourteen (70%) participants were male. The median (interquartile range [IQR]) age was 63.5 (43.5–76.5) years. The median weight and body mass index (BMI) were 84.5 (74.3–97.8) kg and 26.9 (23.4–32.0) kg/m^2^, respectively. The median hematocrit, creatinine, and CrCL were 37.5 (29.3–40)%, 78 (62.3–88) µmol/L, and 108 (71–147) mL/min, respectively. Nineteen (95%) participants received ceftriaxone 2 g daily, and one patient received 2 g 12-hourly. All participants received the full dose of SC ceftriaxone as planned.

### Tolerability and safety

There were no serious adverse events reported during the study. Pain was assessed in 18 participants. Two participants had no abdominal sensation due to previous spinal cord injuries and did not have pain scores assessed. Reported maximum NRS pain scores during and within the 2 hours post-infusion ranged from 0 to 6 (median [IQR] 2.5 [1–4]). All participants were pain-free 4 hours after the infusion. Two participants reported a pain score of 0 throughout the SC infusion and the subsequent 24 hours.

The majority of participants (14 [70%]) had no infusion site erythema during or after the subcutaneous infusion. Five participants had mild erythema (grade 1), and one participant had moderate erythema (grade 2). Erythema had resolved in all but one patient by 24 hours.

A majority of participants (14 [70%]) had some infusion site swelling during and immediately after the infusion. This was scored as grade 1, 2, and 3 in 10, 3, and 1 participants, respectively. By 1 hour, this was only mild in eight and completely resolved beyond 2 hours.

### PK modeling

A total of 212 ceftriaxone DBS concentrations were included in the population PK analysis. The concentration–time profiles were best described by a two-compartment model with first-order absorption and elimination. The estimated PK parameters included total CL, central volume of distribution (Vc), peripheral volume of distribution (Vp), intercompartment clearance (Q), bioavailability from SC administration (Fsc), and the SC absorption rate constant (KAsc). Interindividual variability (IIV) was estimated for CL and Vc, with a proportional error model providing the best fit.

Covariates that significantly reduced the model’s OFV in a stepwise forward process included CrCL on CL, which decreased the OFV by 10.939, with the IIV of Cl decreasing from 51.5% to 45.1%. The inclusion of age on Vc further reduced the OFV by 6.992, while the IIV of Vc decreased from 29.2% to 24%. The final population PK models for CL and Vc were defined as follows: CL = 1.57 × CrCl/100 and Vc = 17.6 × (WT/70) ×exp(0.0094×(AGE-60)).

The model-estimated population PK parameters with their associated inter-individual and residual variabilities for ceftriaxone DBS concentrations, along with bootstrap results, are provided (Table 2). Goodness-of-fit plots of the final population PK model and visual predictive checks with 90% prediction intervals, respectively, are also shown (Fig. S1 and S2). The overall mean residual variability of the model was 15.7%, indicating good model fit.

**TABLE 2:** Final population PK model parameters and bootstrap results for ceftriaxone^*a*^

Parameter	Mean	RSE%	Bootstrap median [95% CI]
Objective function value	1131.055		115.246 [1045.382–1204.996]
Structural model parameters			
CL, l.h^-1^.(100 mL/min)^−1^	1.57	10	1.52 [1.06–1.88]
Vc, l.70kg^−1^ for a 60 years old	17.6	6	17.6 [15.4–20.2]
θ_AGE_ where Vc × EXP(θ_AGE_ *(AGE-60))	0.0094	33	0.0091 [0.0006–0.01471]
V_P_, l.70kg^−1^	12.9	21	12.8 [8.9–48.2]
Q, l.h^−1^.70kg^−**1**^	0.917	12	0.945 [0.692–1.319]
F_**SC**_	0.958	2	0.957 [0.903–0.995]
KA_SC_, h^−**1**^	0.764	13	0.764 [0.579–0.985]
Interindividual variability [shrinkage%]			
IIV CL	45.3 (3)	21	42.6 [25.6–63.8]
IIV V_**C**_	24 (11)	11	22.8 [15.0–28.2]
Residual errors			
Proportional error (%)	15.7 (9)	1	15.5 [13.1–17.8]

^*a*^ CI, confidence interval; CL, population value of renal clearance for an individual with CrCl of 100 mL/min; Vc, population central volume of distribution for an individual with body weight of 70 kg and age 60 years; θAGE, exponent coefficient for age-related adjustment of the volume of distribution; Vp, population peripheral volume of distribution for an individual with a body weight of 70 kg; Q, intercompartment clearance; FSC, absolute subcutaneous bioavailability; KASC, subcutaneous absorption rate constant; % RSE, relative standard error [%RSE = 100 * (standard error/parameter estimate)]; inter-individual variability (IIV) and residual error are presented as 100% × √variability estimate.

The estimated bioavailability for SC ceftriaxone was 95.7% (95% bootstrap interval 90.3–99.5). Weight and BMI did not have any influence on the absorption rate from the SC space into the central compartment.

**Fig 2:**
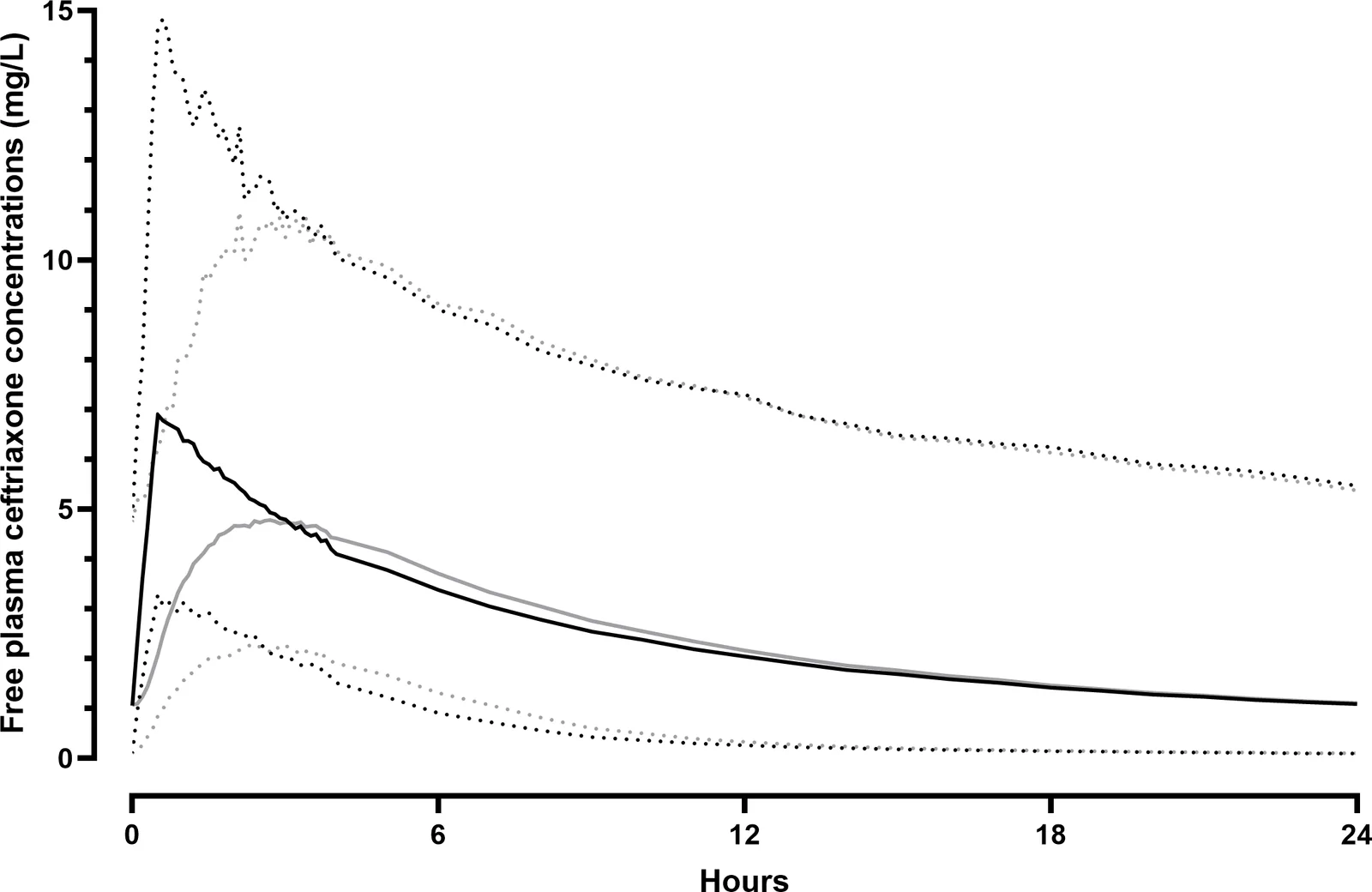
Simulated mean steady-state unbound concentrations of ceftriaxone over 24 hours. Standard IV dosing (2 g daily, black, with 95% confidence intervals; dotted line) is compared with the same dose given SC (gray, with 95% confidence intervals; dotted line).

**Fig 3:**
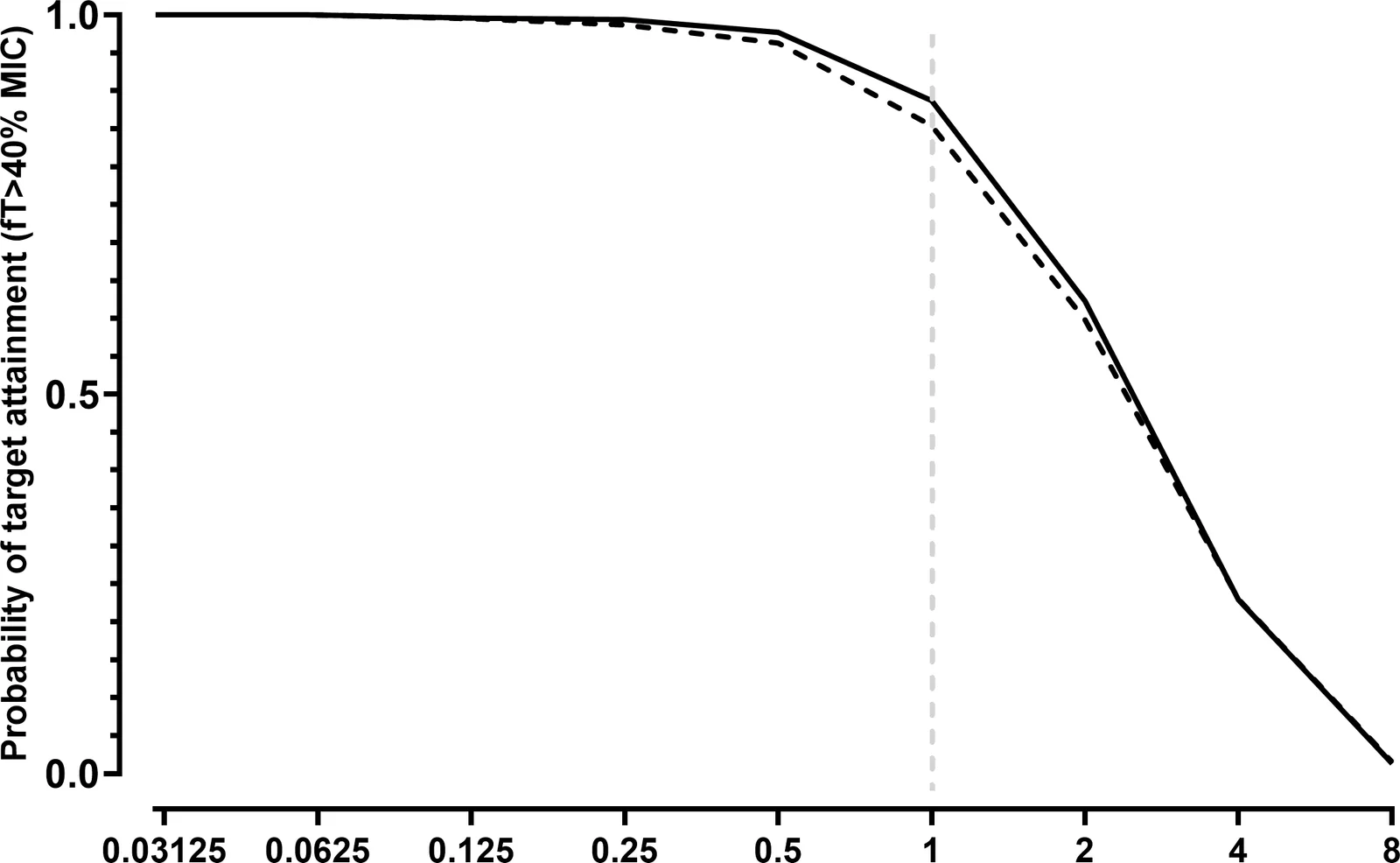
Probability of target attainment, defined by unbound ceftriaxone concentrations exceeding the MIC for 40% of the time between injections at steady state, according to possible MICs. Standard IV dosing (2 g, dashed line) is compared with the same dose given SC (solid line). The breakpoint for *Streptococcus pneumoniae* and Enterobacterales is shown (1 mg/L; gray vertical line).

Using the final model, simulations demonstrated that ceftriaxone delivered SC had lower peak and comparable trough concentrations relative to IV dosing (Fig. 2). Furthermore, the PTA (fT > 40%) at MIC between 0.03 and 1 mg/L was comparable following SC administration (Fig. 3).

## DISCUSSION

SC delivery of antibiotics is an alternative route of administration in scenarios where IV administration is challenging. In this study, we demonstrated that SC infusion of a 2 g dose of ceftriaxone appears to be safe and well tolerated. Although most patients had mild pain and some subcutaneous swelling during or immediately after the infusion, pain and swelling had completely resolved in all participants 4 hours post-infusion. When compared with IV administration, peak concentrations are lower with similar trough concentrations, resulting in a comparable PTA for common pathogens causing community-acquired pneumonia, urinary, and intra-abdominal infections. The bioavailability of SC administration was ~95%.

These data extend the findings of literature on SC ceftriaxone delivered as a 1 g dose. The safety of a higher dose is of particular interest because 2 g is now the standard recommendation in many guidelines (1, 17–19). As our study recruited hospitalized patients already receiving ceftriaxone for the management of infection, these data should be generalizable to non-critically ill patients with a variety of severe infections, in contrast with studies recruiting healthy volunteers (26, 27).

There are several scenarios where SC administration of ceftriaxone 2 g might be advantageous.

First, in settings where parenteral medications are still required but IV access is difficult to obtain. This might be of particular relevance to smaller hospitals where ultrasound guidance or senior expertise may not be available after hours. Second, in patients requiring prolonged parenteral therapy, SC administration could reduce the complications and costs associated with long-term vascular access devices. Our study cohort included individuals with liver abscesses, infected intra-abdominal collections, prosthetic joint infections, and prosthetic valve infective endocarditis. These patients required prolonged courses of antibiotics, and in this setting, SC administration is an attractive alternative to repeated peripheral cannulation or peripherally inserted central catheter (PICC) line insertion that may also enable more timely discharge from hospital. Indeed, due to ceftriaxone’s safety profile, convenient dosing, and extensive experience in ambulatory settings, SC administration could be rapidly adopted by OPAT programs to facilitate parenteral antibiotic delivery in situations where ongoing intravenous access is challenging or not desired. Finally, specific cohorts including patients with chronic kidney disease, in which vein preservation strategies are an important consideration, might benefit from the routine use of SC administration of antibiotics (28).

Inclusion of age as a covariate improved estimation of volume of distribution, and it is likely that age is providing a surrogate measure of frailty (29). Optimization of ceftriaxone dosing strategies in the frail elderly deserves further exploration. Most guidelines do not recommend dosage adjustment in the context of renal function impairment (30); however, inclusion of creatinine clearance improved estimation of clearance within the model. Noting that the study population mostly had normal or mild impairment in renal function, further study in patients with chronic kidney disease should be conducted, including exploration of extended dosing intervals. Hypoalbuminemia in the context of critical illness and systemic inflammation is associated with increased free ceftriaxone levels, which may increase clearance (31). Albumin was not included in our final model, but it should be noted that our cohort did not include many patients with significant hypoalbuminemia, and no participants had severe liver disease, so the model should not be extrapolated to these groups. However, in each of these special populations, we would expect similar effects to pharmacokinetics of both SC and IV ceftriaxone.

Our study only included adult patients who were clinically stable, and these data may not be applicable to patients with critical illness where PK/PD targets and absorption from the SC tissue may be different. Further work is required to demonstrate the safety and tolerability in other patient groups, including children. As we only observed a single dose, tolerability to repeated infusions is not guaranteed, and evaluating the tolerability of multiple doses should be explored further.

### Conclusions

SC administration of 2 g ceftriaxone by intermittent infusion appears to be well tolerated with excellent bioavailability and comparable PTA to IV administration in non-critically ill patients with severe infections.

## SUPPLEMENTAL MATERIAL

Supplemental Material aac.01081-25-s0001.docx Table S1; Fig. S1 to S4.

ASM does not own the copyrights to Supplemental Material that may be linked to, or accessed through, an article. The authors have granted ASM a non-exclusive, world-wide license to publish the Supplemental Material files. Please contact the corresponding author directly for reuse.

## References

[1] van de Beek D, Cabellos C, Dzupova O, Esposito S, Klein M, Kloek AT, Leib SL, Mourvillier B, Ostergaard C, Pagliano P, Pfister HW, Read RC, Sipahi OR, Brouwer MC, ESCMID Study Group for Infections of the Brain (ESGIB). 2016. ESCMID guideline: diagnosis and treatment of acute bacterial meningitis. Clin Microbiol Infect 22 Suppl 3:S37–S62. doi:10.1016/j.cmi.2016.01.007 27062097

[2] Kranz J, Bartoletti R, Bruyère F, Cai T, Geerlings S, Köves B, Schubert S, Pilatz A, Veeratterapillay R, Wagenlehner FME, Bausch K, Devlies W, Horváth J, Leitner L, Mantica G, Mezei T, Smith EJ, Bonkat G. 2024. European association of urology guidelines on urological infections: summary of the 2024 guidelines. Eur Urol 86:27–41. doi:10.1016/j.eururo.2024.03.035 38714379

[3] SA Health. 2024. National antimicrobial utilisation surveillance program: 2022 annual report. Department of Health and Aged Care, Canberra.

[4] Gauthier D, Schambach S, Crouzet J, Sirvain S, Fraisse T. 2014. Subcutaneous and intravenous ceftriaxone administration in patients more than 75 years of age. Med Mal Infect 44:275–280. doi:10.1016/j.medmal.2014.03.007 24932703

[5] Kalatharan L, Ferman M, Kumar S, Rajendra S, Pripanapong S, Wu Y, Richards H, Rogers BA. 2023. Use of ceftriaxone and benzylpenicillin in outpatient parenteral antimicrobial therapy: spectrum vs cost. Open Forum Infect Dis 10:ofad505. doi:10.1093/ofid/ofad505 37965641 PMC10641299

[6] Kevat PM, Reeves BM, Ruben AR, Gunnarsson R. 2017. Adherence to secondary prophylaxis for acute rheumatic fever and rheumatic heart disease: a systematic review. Curr Cardiol Rev 13:155–166. doi:10.2174/1573403X13666170116120828 28093988 PMC5452151

[7] Roubaud-Baudron C, Forestier E, Fraisse T, Gaillat J, de Wazières B, Pagani L, Ingrand I, Bernard L, Gavazzi G, Paccalin M. 2017. Tolerance of subcutaneously administered antibiotics: a French national prospective study. Age Ageing 46:151–155. doi:10.1093/ageing/afw143 28181635

[8] Goutelle S, Valour F, Gagnieu M-C, Laurent F, Chidiac C, Ferry T, Lyon Bone and Joint Infection Study Group. 2018. Population pharmacokinetics and probability of target attainment of ertapenem administered by subcutaneous or intravenous route in patients with bone and joint infection. J Antimicrob Chemother 73:987–994. doi:10.1093/jac/dkx477 29244077

[9] Murray F, Yoo O, Brophy-Williams S, Rawlins M, Wallis SC, Roberts JA, Raby E, Salman S, Manning L. 2025. Safety, tolerability and pharmacokinetics of subcutaneous cefazolin as an alternative to intravenous administration. J Antimicrob Chemother 80:347–353. doi:10.1093/jac/dkae397 39671325 PMC11787891

[10] Murray F, Yoo O, Brophy-Williams S, Rawlins M, Wallis SC, Roberts JA, Raby E, Salman S, Manning L. 2025. Safety, tolerability and pharmacokinetics of subcutaneous meropenem as an alternative to intravenous administration. J Antimicrob Chemother 80:209–215. doi:10.1093/jac/dkae398 39526935

[11] Colin E, Baldolli A, Verdon R, Saint-Lorant G. 2020. Subcutaneously administered antibiotics. Med Mal Infect 50:231–242. doi:10.1016/j.medmal.2019.06.007 31300245

[12] Hernández-Ruiz V, Forestier E, Gavazzi G, Ferry T, Grégoire N, Breilh D, Paccalin M, Goutelle S, Roubaud-Baudron C, GInGer (Groupe Infectio-Gériatrie). 2021. Subcutaneous antibiotic therapy: the why, how, which drugs and when. J Am Med Dir Assoc 22:50–55. doi:10.1016/j.jamda.2020.04.035 32674952

[13] Kado JH, Salman S, Henderson R, Hand R, Wyber R, Page-Sharp M, Batty K, Carapetis J, Manning L. 2020. Subcutaneous administration of benzathine benzylpenicillin G has favourable pharmacokinetic characteristics for the prevention of rheumatic heart disease compared with intramuscular injection: a randomized, crossover, population pharmacokinetic study in healthy adult volunteers. J Antimicrob Chemother 75:2951–2959. doi:10.1093/jac/dkaa282 32696033

[14] Stoner KL, Harder H, Fallowfield LJ, Jenkins VA. 2014. Intravenous versus subcutaneous drug administration. which do patients prefer? A systematic review. Patient. doi:10.1007/s40271-014-0075-y 25015302

[15] Borner K, Lode H, Hampel B, Pfeuffer M, Koeppe P. 1985. Comparative pharmacokinetics of ceftriaxone after subcutaneous and intravenous administration. Chemotherapy 31:237–245. doi:10.1159/000238342 4028870

[16] Pouderoux C, Becker A, Goutelle S, Lustig S, Triffault-Fillit C, Daoud F, Fessy MH, Cohen S, Laurent F, Chidiac C, Valour F, Ferry T, Lyon Bone and Joint Infection Study Group. 2019. Subcutaneous suppressive antibiotic therapy for bone and joint infections: safety and outcome in a cohort of 10 patients. J Antimicrob Chemother 74:2060–2064. doi:10.1093/jac/dkz104 31220276

[17] Community acquired pneumonia in adults. 2025. Therapeutic guidelines. Melbourne Therapeutic Guidelines Limited. https://www-tg-org-au.qelibresources.health.wa.gov.au.

[18] Peritonitis due to perforated viscus. 2025. Therapeutic guidelines. Melbourne Therapeutic Guidelines Limited. https://www-tg-org-au.qelibresources.health.wa.gov.au.

[19] Directed therapy for native bone or joint infection. 2025. Therapeutic guidelines. Melbourne Therapeutic Guidelines Limited. https://www-tg-org-au.qelibresources.health.wa.gov.au.

[20] Administration USFaD. 2021. Bioequivalence studies with pharmacokinetic endpoints for drugs submitted under an abbreviated new drug application. FDA Maryland.

[21] Page-Sharp M, Nunn T, Salman S, Moore BR, Batty KT, Davis TME, Manning L. 2016. Validation and application of a dried blood spot ceftriaxone assay. Antimicrob Agents Chemother 60:14–23. doi:10.1128/AAC.01740-15 26438505 PMC4704204

[22] Mukap M, Sprod C, Tefuarani N, Laman M, Page-Sharp M, Salman S, Moore BR, Batty KT, Davis TME, Manning L. 2018. Validation of a dried blood spot ceftriaxone assay in Papua New Guinean children with severe bacterial infections. Antimicrob Agents Chemother 62:e00940-18. doi:10.1128/AAC.00940-18 30012775 PMC6153847

[23] Bijur PE, Latimer CT, Gallagher EJ. 2003. Validation of a verbally administered numerical rating scale of acute pain for use in the emergency department. Acad Emerg Med 10:390–392. doi:10.1111/j.1553-2712.2003.tb01355.x 12670856

[24] Keizer RJ, Karlsson MO, Hooker A. 2013. Modeling and simulation workbench for NONMEM: tutorial on Pirana, PsN, and Xpose. CPT Pharmacometrics Syst Pharmacol 2:e50. doi:10.1038/psp.2013.24 23836189 PMC3697037

[25] CLSI. 2024. Performance standards for antimicrobial susceptibility testing. In CLSI guideline M100, 34th ed. Clinical and Laboratory Standards Institute.

[26] Harb G, Lebel F, Battikha J, Thackara JW. 2010. Safety and pharmacokinetics of subcutaneous ceftriaxone administered with or without recombinant human hyaluronidase (rHuPH20) versus intravenous ceftriaxone administration in adult volunteers. Curr Med Res Opin 26:279–288. doi:10.1185/03007990903432900 19947907

[27] Shearer TW. 2016. Pharmacokinetic response after sub-cutaneous administration of ceftriaxone. IDWeek, New Orleans, USA.

[28] Vachharajani TJ, Hassanein M, Liaqat A, Haddad N. 2020. Vessel preservation in chronic kidney disease. Adv Chronic Kidney Dis 27:177–182. doi:10.1053/j.ackd.2020.03.006 32891300

[29] Tan SJ, Cockcroft M, Page-Sharp M, Arendts G, Davis TME, Moore BR, Batty KT, Salman S, Manning L. 2020. Population pharmacokinetic study of ceftriaxone in elderly patients, using cystatin C-based estimates of renal function to account for frailty. Antimicrob Agents Chemother 64:e00874-20. doi:10.1128/AAC.00874-20 32778543 PMC7508575

[30] Therapeutic guidelines. 2025. Melbourne Therapeutic Guidelines Limited. https://www-tg-org-au.qelibresources.health.wa.gov.au.

[31] Michel J, Monti F, Lamoureux F, Diagouraga D, Etienne M, Quillard M, Molkhou C, Tamion F, Dahyot S, Petersen T, Pereira T, Pestel-Caron M, Grosjean J, Duflot T. 2025. Unraveling ceftriaxone dosing: free drug prediction, threshold optimization, and model validation. AAPS J 27:50. doi:10.1208/s12248-025-01041-w 40011393

