# Supplementary material for: Safety, tolerability, and pharmacokinetics of a 2 g subcutaneous dose of ceftriaxone as an alternative to intravenous delivery
Source: Antimicrob Agents Chemother. 2025 Nov 24;70(1):e01081-25. doi: 10.1128/aac.01081-25 (PMC12777559; doi:10.1128/aac.01081-25)

**Supplementary Data**

Table S1 – Exclusion Criteria

| **Exclusion Criteria** |
| --- |
| - Patients not clinically stable, as defined by being in ICU, or having had a MET call in the 24 hours before screening for enrolment. - Children <18 years - Patients whose treating team predict that ceftriaxone will be ceased within 48 hours. - History of anaphylaxis or serious adverse reaction to cephalosporin in the past. - Patients unable to give informed consent themselves. |

**Supplementary Figure 1.** Goodness-of-fit (GOF) plots for the IV and SC ceftriaxone infusion final model. (A) Individual predicted concentration versus observed concentration; (B) Population-predicted concentration versus observed concentration; (C) Conditional weighted residuals (CWRES) versus Population-predicted concentration; (D) CWRES versus time; The black lines in (A) and (B) show the line of identity. The black lines in (C) and (D) show the zero reference line.


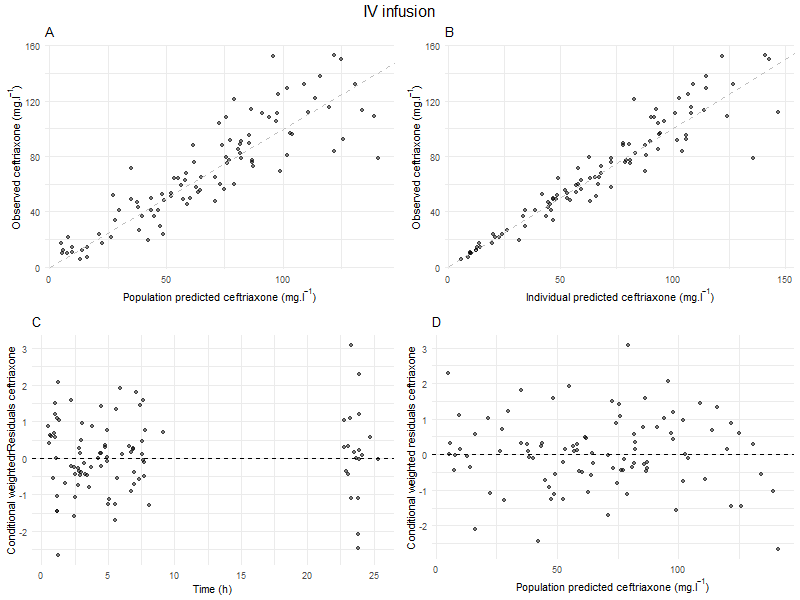


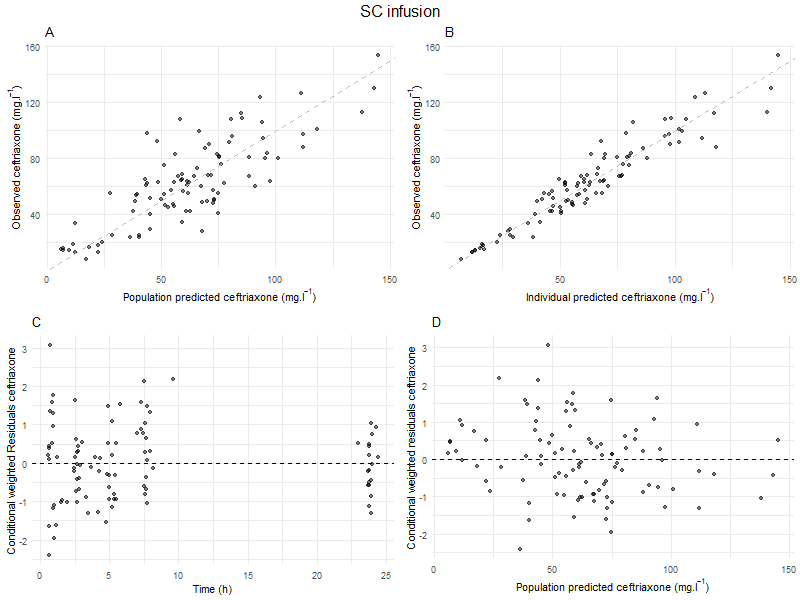


**Supplementary Figure 2.** Visual Predictive Checks for DBS ceftriaxone concentrations in plasma after IV and SC infusions: Dosing via IV infusion (A); Dosing via SC infusion (B). Open circles represent the measured concentrations of total ceftriaxone. Solid lines depict the median values of the observed data. Dashed lines indicate the 5th and 95th percentiles of the observed data. Shaded areas illustrate the 95% confidence intervals for the simulated values from the pharmacokinetic model, with: Upper and lower shaded areas corresponding to the 95th and 5th percentiles, respectively; Middle shaded area representing the 50th percentile. Concentration is expressed in mg/L.


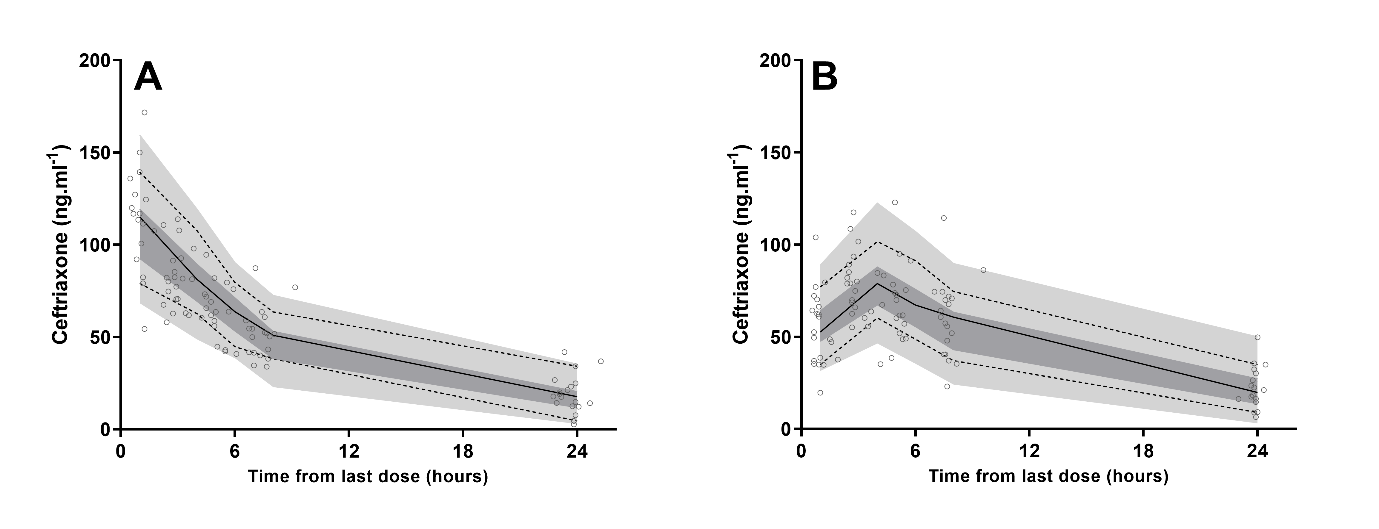


**Supplementary Figure 3**. Observed ceftriaxone concentrations (µg/mL) versus time (hours) by administration route. Each panel shows individual subject profiles, with colors distinguishing subjects. Intravenous (IV) dosing is displayed on the left panel and subcutaneous (SC) dosing on the right panel.


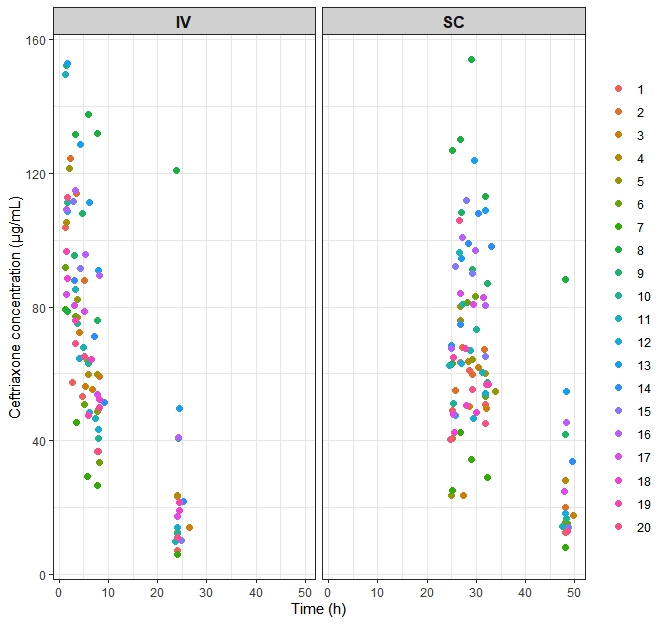


**Supplementary Figure 4**. Extended simulations of unbound concentrations over time, starting from the first dose through to steady state.


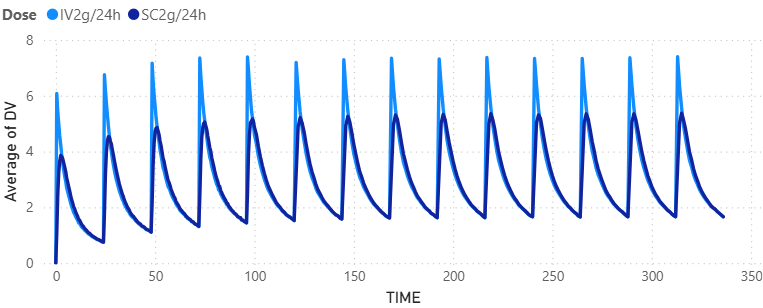

Supplement: Supplemental Material — Table S1; Fig. S1 to S4. [file aac.01081-25-s0001.docx]
